# Precision public health to inhibit the contagion of disease and move toward a future in which microbes spread health

**DOI:** 10.1186/s12879-019-3715-y

**Published:** 2019-02-06

**Authors:** David S. Thaler, Michael G. Head, Andrew Horsley

**Affiliations:** 1 Biozentrum, University of Basel, Klingelbergstrasse 50/70, CH-4056 Basel, Switzerland; 2 Clinical Informatics Research Unit, Faculty of Medicine, University of Southampton, University Hospital Southampton, Coxford Road, Southampton, SO16 6YD UK; 3 Research School of Physics and Engineering, The Australian National University, Mills Rd., Canberra, ACT 2601 Australia

**Keywords:** Precision medicine, Vaccines, Infectious vaccines, Public health, Precision public health, Infectious disease, Epidemiology, Bioethics, Microbiome, Healthy buildings

## Abstract

Antimicrobial resistance continues to outpace the development of new chemotherapeutics. Novel pathogens continue to evolve and emerge. Public health innovation has the potential to open a new front in the war of “our wits against their genes” (Joshua Lederberg). Dense sampling coupled to next generation sequencing can increase the spatial and temporal resolution of microbial characterization while sensor technologies precisely map physical parameters relevant to microbial survival and spread. Microbial, physical, and epidemiological big data could be combined to improve prospective risk identification. However, applied in the wrong way, these approaches may not realize their maximum potential benefits and could even do harm. Minimizing microbial-human interactions would be a mistake. There is evidence that microbes previously thought of at best “benign” may actually enhance human health. Benign and health-promoting microbiomes may, or may not, spread via mechanisms similar to pathogens. Infectious vaccines are approaching readiness to make enhanced contributions to herd immunity. The rigorously defined nature of infectious vaccines contrasts with indigenous “benign or health-promoting microbiomes” but they may converge. A “microbial Neolithic revolution” is a possible future in which human microbial-associations are understood and managed analogously to the macro-agriculture of plants and animals. Tradeoffs need to be framed in order to understand health-promoting potentials of benign, and/or health-promoting microbiomes and infectious vaccines while also discouraging pathogens. Super-spreaders are currently defined as individuals who play an outsized role in the contagion of infectious disease. A key unanswered question is whether the super-spreader concept may apply similarly to health-promoting microbes. The complex interactions of individual rights, community health, pathogen contagion, the spread of benign, and of health-promoting microbiomes including infectious vaccines require study. Advancing the detailed understanding of heterogeneity in microbial spread is very likely to yield important insights relevant to public health.

## Background public health in the pre and the post antibiotic eras

**Fig. 1:**
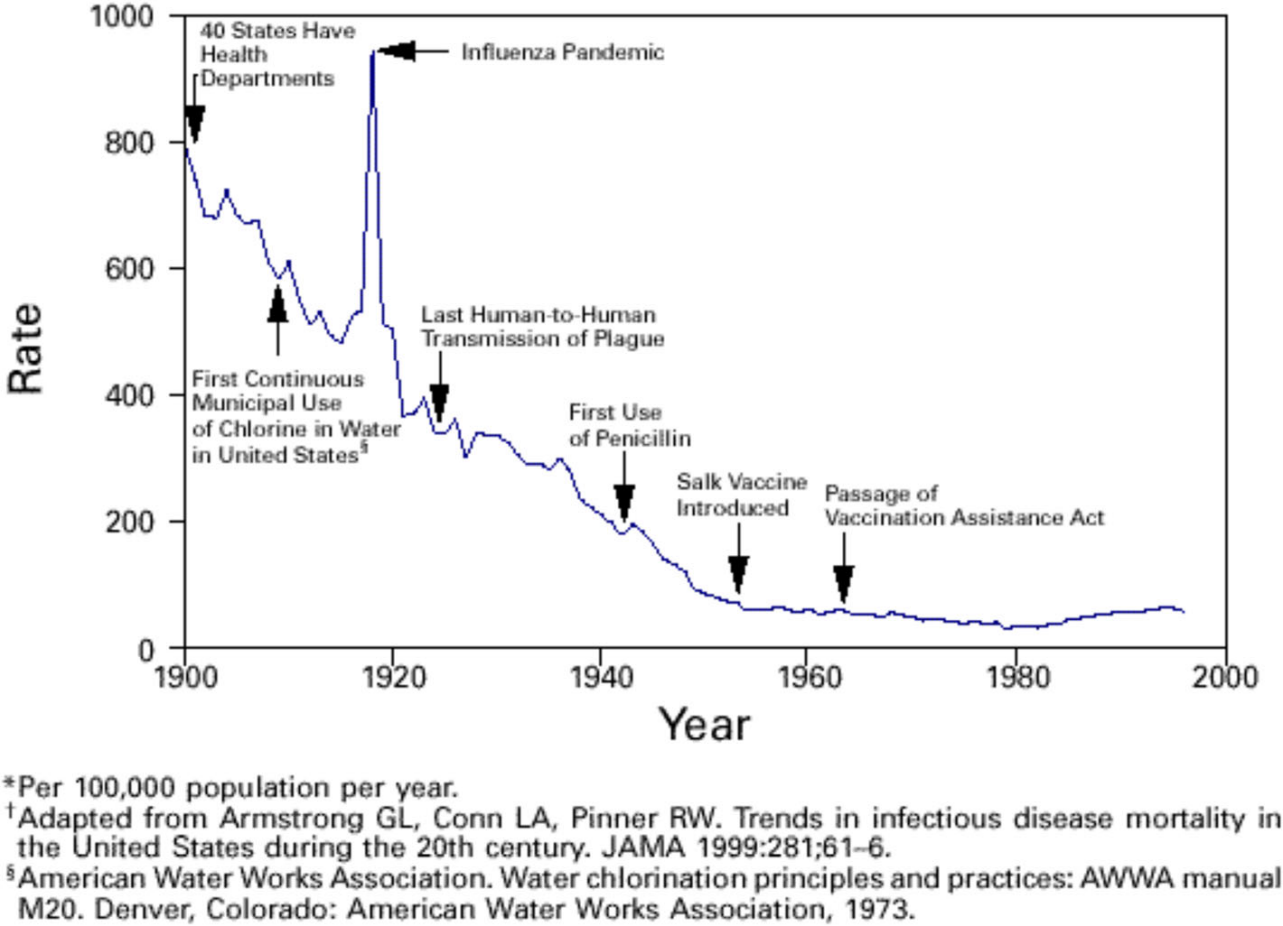
Crude death rate for infectious diseases – United States, 1900–1996 (adapted by the CDC from [112]). Mortality from infectious diseases began to decrease before the advent of antimicrobials. The slope of decrease in crude mortality does not show evidence of being affected by the introduction of antimicrobials. The first clinical use of sulfonamids was in 1935, penicillin in 1943, with others throughout the following decades, albeit with decreasing frequency of novel compounds. The 1918 spike in mortality was caused by the Spanish flu pandemic which is estimated to have infected 500 million people, one third of the world’s population at that time, and killed between twenty and fifty million people

The antibiotic era began in the 1940’s. A trend toward decreasing mortality from infectious diseases began fifty years earlier in the late nineteenth century [1]. At least three quarters of the decrease in mortality from infectious disease from 1900 to the present day era appears due to public health measures because antibiotics became significant only in the 1940’s (see Fig. 1). Resistances to antimicrobials are increasingly common and threaten an end of the era of reliable treatment. It is “our wits against their genes” without a clear advantage to the former [2]. New antimicrobials will be discovered and developed but new resistances will arise. The continuing power of antimicrobials is at risk and alternatives must be actively considered [3, 4]. Personalized and/or Precision public health could fit under already-existing rubrics of prevention via surveillance and sanitation. Alternatively, these approaches may be considered radical and different enough to suggest Personal and Precision Public Health as including novel elements and emphases.

The meaning and scope of the terms Personalized and/or Precision Public Health are a subject of current debate [5–9]. There is controversy about what Precision Public Health encompasses, how new the parts are, and what value they may or may not have. Some differences in the present article compared to other expositions should be noted. Here we: 1) Emphasize monitoring physical characteristics in ‘smart buildings’ 2) De-emphasize the contribution of human genomic DNA sequences. As a related remark, susceptibility to infectious diseases is more likely to have a meaningful and robust genetic component in the young than in adult or aged individuals [10]. 3) Emphasize finding the optimum granularity. By “granularity” we mean the size, time, or other divisions for which heterogeneity is most meaningful. We hypothesize the individual human being as likely to represent an especially meaningful level of granularity with regard to the microbial environment. 4) Future-oriented scenarios that anticipate Precision Public health promoting the spread of health-promoting microbiomes including, but not limited to, infectious vaccines.

## Heterogeneity in biology

Technical innovations that allow higher resolution scrutiny have led to progress- even revolutions- in many subfields of biology. Examples include dividing tissues into cellular subtypes [11], single cell expression profiles from defined anatomy, cell types, or developmental lineages [12–14], and cells themselves fractionated into organelles [15]. An analogous revolution in public health might be possible through higher resolution mapping of relevant parameters.

This commentary focuses on technologies for, and the consequences of, increased spatial and temporal resolution of microbiological, chemical, physical, spatial and temporal measurements relevant to public health. There is a “chicken and egg” or “Catch 22” problem in anticipating the benefits of higher-resolution data. This limitation follows from our current ignorance of how heterogeneous the relevant physical and microbiological parameters are. New insights and benefits are likely to be proportional to how much heterogeneity is discovered as a consequence of gains in resolution. To begin research that might crack the chicken and egg problem, one must initially speculate on the relevant heterogeneity. By hypothesis, a relevant unit of heterogeneity to consider for airborne infection will be at the level of each individual human being. The scales at which heterogeneity is hypothesized to exist affect the research programs to search for it. The results will subsequently be used to modify the hypotheses and research program. Heterogeneity will be discussed further in following sections.

The technical means and value of increasing spatial and temporal resolution in other areas of public health, such as ensuring healthy drinking water [16] and optimal transformation of sewage [17] or compost, [18] will not be covered here.

The human exposome is currently defined elsewhere as the sum of an individual’s lifetime exposure to microbes and chemicals [19]. We suggest that this definition of the human exposome misses vital elements of what matters and the definition could usefully be extended in two related ways: 1) It should explicitly include microbes and chemicals sourced directly from other humans which probably are a major contributor in many circumstances and 2) The exposome should be understood as a reciprocal and interactive process to account for the fact that each individual is both a source and a sink of microbes and chemicals. The current definition of the exposome seems to consign a role to individual humans as passive recipients or victims of their environment. This is a valid way to model parts of the system, including the political system, but it is biologically an incomplete description of the consequences of our being social animals. Individual humans are major microbiological and probably chemical contributors to their immediate environment. Humans in proximity are exposed to each other’s microbiomes and chemicals. Some of this is classical epidemiology of infectious disease, such as transmission of human-specific viruses including influenza. Some chemical influences might be classed as behavioral, e.g. cigarette smoke and the experience of second hand smoke. However, it is also plausible that significant chemicals that modify the exposome of self and of others arise via the microbiome or other metabolic sources over which an individual has, at present, no means of knowledge or control.

## Mapping what matters for human-relevant microbes and microbiomes

Tang et al. propose more precisely mapping the global epidemiology of non-influenza RNA respiratory viruses, based on the whole genome sequencing of these viruses [20]. We are supportive of this and other related large-scale data-mining projects, i.e. with the aim that such efforts yield copious and interpretable data. Information currently undiscovered might also be obtained using a different approach to sampling, particularly air-sampling.

Current air sampling strategies concentrate microbes from larger volumes (liters to hundreds cubic meters) of air into smaller volumes of liquid (either directly or from filters that are extracted) [21]. Concentration economizes the use of reagents and may (depending on inhibitors and dilution effects) minimize the frequency of assays that yield no signal. However, vacuum sampling alter airs flow making it a biological instantiation of the Heisenberg uncertainty principle whereby the act of measurement affects the object of measurement. This sampling approach makes a trade-off between sensitivity and localization.

How much heterogeneity is there in the spatial and temporal distribution of microbes in the built environment? Airborne fungi in different rooms vary across seasons in residences [22]. The microbial exposure where infants crawl and young children toddle is distinct from that at the level where adults are breathing [23, 24]. In a case study reported in this issue, Tang et al. find that individual samplers on different individuals varied from zero RSV RNA copies to a high of 2778/m^3^. It is currently unknown how much heterogeneity is inside the cubic meter that contains thousands of genomes. At the extremes, the entire viral load could have been confined to one compact particle or, alternatively, it could have been evenly dispersed through the entire volume.

Sequences or detection alone are not the only informational product. The usefulness of spatial and temporal metadata depends on how much they reveal heterogeneity. PCR can now be carried out inside nanoliter or picoliter volumes with many millions carried out in parallel and rare hits separated by Florescence Activated Cell Sorting (FACS) from a majority of negative droplets [25]. Single molecule sequencing is becoming routine and molecules can uniquely labeled with barcoded primers. What remains to be invented are ways to combine barcoding with nanodrop assays and single molecule sequencing such that each molecule’s sequence or nanoliter drop PCR assay can be linked to its unique metadata. Small droplet assays are likely to decrease background due to contamination of molecular reagents with bacterial DNA [26].

Rapid and focused public health actions motivated by precision data could lead to less overall disruption and more efficient prevention [27]. For example, if infected animals can be reliably identified, there may be less need to cull larger groups. Individual cattle, pigs and chicken cages are already identified in current agricultural settings to allow the tracing of Bovine Spongiform Encephalopathy (BSE) [28]. Globally, approximately 20% of livestock are lost to preventable diseases, costing around USD $2 billion per annum in Africa alone, with much of this loss in low- and lower-middle income settings [29]. There is substantial potential for veterinary, human public health and economic benefit.

### Toward improved microbial sampling in the built environment

A widely cited survey estimated that most people in the US spend 90% or more of their time either inside buildings or automobiles [30]. Similar considerations apply in buildings and more broadly for example ships, subways, and airplanes. Most potentially consequential microbial exposure probably occurs in these venues [31]. There are three sources of microbes in buildings [32]: a) occupants, i.e. people and other animals if present b) penetration from the outside environment c) growth in/on the structure itself e.g. walls, pipes and on surfaces that are intermittently wet [33]. Microbial growth in garbage, among foodstuffs, on furnishings and cloths contributes in some cases [34]. The contribution of each source to the medically relevant microbiome is not equally distributed. Most airborne viruses that infect humans spread from other humans although zoonotic transmission in farms and markets should not be overlooked.

### Heterogeneity and sampling

The optimum mode of sampling depends on the distribution of targets. If the target is rare and evenly distributed, then gathering as much of the environment as possible into each sample is best. However, if the target is unevenly distributed, then smaller sample sizes may be better so that rare targets are not diluted below the detection threshold. If the environment or reagents also contain inhibitors or decoy targets, then large scale concentration may decrease sensitivity. Taq polymerase and many other reagents contain bacterial DNA that is residual from their production. Assays that use minimal amounts of Taq decrease this background [26] and small volumes assays should further help.

For the detection of Biowarfare agents that are normally absent and for which quick, specific, and sensitive detection are paramount, many thousands of liters of air rapidly collected are appropriate [35]. On the other hand, to best understand the undisturbed environment the sampling method should be gentle, not itself create additional airflow, and it should be granular. Individual vacuum samplers are an intermediate methodology. They are worn or left near a patient and draw air onto a filter. Depending on the airflow rate they sample a variable-sized environment. The time resolution is typically one to several hours. As currently used the whole filter, which is typically 25 mm in diameter is extracted as a single sample [36, 37].

Insight into heterogeneity of the air might be gained through a modification of the way that the sampling filter is currently processed. Instead of extracting the filter in one piece, suppose it were cut into a number of equal segments that are processed and assayed independently. If most segments are negative but a few contain a strong signal that would be clear indication of heterogeneity in the air volume sampled. This reasoning and experimental design take their inspiration from classic studies that determined the independence of bacterial mutation from its subsequent selection [38, 39].

### Individual passive sampling

If spatial and temporal variation are high, then smaller more localized and less mixed samples become relatively advantageous. The mouth and nose are the expected source and the reservoir for most airborne infectious agents. Dilution into the air volume predicts that the concentration of an airborne agent will decrease as a cubic function of distance from its source. Deactivation of viruses in aerosol may lead to a more dramatic drop-off of viable virus. Samplers should be unobtrusive and not alter the sampled environment. The assay of tiny volumes and the ability isolate rare positive samples recommend a new generation of small volume and/or passive samplers.

Clothing is a promising personal environmental sampling site [40, 41] but difficult to standardize. One might build on the clothing idea to design defined samplers optimized for capture and preservation of the microbial and chemical environment. Sampling material might be housed in specially-designed holders, akin to radiation badges. For example, in hospitals, all patients, staff and visitors might wear standardized samplers.

The top of the shoulders appears to offer a minimally obtrusive yet maximally sensitive sampling site. Top-of-the-shoulder samplers would be closer to the mouth and nose than samplers worn as chest badges but relative sensitivity remains to be tested. Epaulettes such as those worn as military insignia might provide design inspiration. These approaches would potentially dovetail with simultaneously tracking movements within the environment of interest, and thus early consideration of the ethical issues and public or user concerns would be critical.

Passive samplers have different characteristics that in some scenarios may make them superior to vacuum-enhanced air sampling: i) if the target distribution is highly heterogenous in space, then source proximity may give the highest signal to noise ratio. ii) the related ability to identify the source with less distortion due to the sampler’s altering airflow. Each sampling patch could contain internal standards. Research to design samplers might take inspiration from materials for replica plating of bacteria [39, 42]. There may even be specific overlap of optimal materials such as velveteen (a velvet-like cloth) for capture.

### Prospective identification of super-spreaders

Super-spreaders or super-shedders are individuals who disproportionately infect others and may dominate the epidemiology of infectious disease [43–46]. In some contexts super-spreaders of infectious agents might be identified prospectively by molecular criteria rather than by *post facto* epidemiology [47, 48]. Asymptomatic super-spreaders of airborne infections might be identified as those whose samplers harbor the most signal for the agents in question [48]. Super-spreaders or super-shedders have been documented in people and also among cattle and in mouse model systems [49, 50].

There are important unknowns concerning super-spreaders. A study in cattle argues that super-shedding may be time rather than individual dependent, i.e. the same individuals may not be super-spreaders a mere few hours later [51]. It is currently unclear- in fact the question appears to be unasked- whether super-spreaders are agent-specific or generalized. By further hypothesis generalized super-spreaders might excrete more of their own DNA as well as that of infectious agents. In this case the detection of spreading potential might be seen in two ways:

i) by the presence of more self-sequences on the individual’s samples and ii) by the presence of individual sequence-specific DNA from the sampling of others. The anticipation would be that those who shed either the “wrong”, i.e. potential pathogen, or perhaps simply too much, DNA may be considered candidates for a variety of control measures, up to and including confinement.

Rational discussions about policy and human rights should be informed by facts [52]. Not all infections spread between individuals are harmful or neutral. Health-positive microbial spreading, e.g. the live attenuated oral polio and rotavirus vaccines, also occurs. Analogous to the way that some individuals are more influential than others in spreading ideas or memes [53], some may be more influential in spreading health-promoting microbiomes. If social and environmental transmission of benign and of positively helpful microbiomes turns out to be the case, will it follow the same rules as the spread of detrimental infections? Depending on their resident microbiomes, the same individuals may turn out to be super-spreaders of benign, health-promoting, or harmful microbes.

### Toward mapping the 3D topography of relevant variables

The survival and distribution of airborne infectious agents depends on temperature, humidity [54–60] and airflow [61, 62]. Improved mapping of these parameters would better inform building design, and real-time monitoring of these parameters in occupied buildings might open the way for their dynamic optimization. For example, there is generally a temperature difference between indoor and outdoor environments, which results in a temperature gradient across rooms whose windows or walls form a building’s exterior. Regions of every intermediate temperature and relative humidity will exist when the outside is below freezing and the inside is warm. Temperature gradients necessarily traverse the dew point where water vapor condenses into liquid. The stability of regions where water vapor liquifies, and the consequences for microbes are complex and situation-specific. Moreover, most things microbial in the built environment also depend on the behavior of occupants. It is therefore challenging for modelling to predict, for example, that a specific region of hospital room, such as the southeast corner near the ceiling, happens to be in a “sweet spot” for bacteria or virus.

An increasing number of ‘smart’ buildings, including hospitals, are fitted with sensors [63–65] which monitor temperature and/or humidity at a given position and are networked in an Internet of Things (IoT) [66, 67]. These IoT systems can provide 3D maps of the monitored parameters in real-time, and can be used e.g. to optimize heating, ventilation, and air conditioning (HVAC) systems [68, 69] or to monitor the integrity of the building structure [70]. Such IoT systems could be readily adapted for applications targeting the microbiome, with additional sensors placed in at-risk areas. Sensors can be installed during building construction, retrofitted permanently, or temporarily installed. The IoT systems could be augmented with other sensors to monitor e.g. levels of particular gas types or volatile organic compounds (VOC) relevant to microbial growth [71–73]. Environmental sensing will interface with wearable technology and individual-identified information [74].

In addition to the temperature and humidity information provided by an IoT network of sensors, there is a need for devices that provide portability, remote sensing, and high spatial resolution. These could be used to rapidly assess large areas without having to install an IoT system, monitor targets otherwise difficult to access, or provide the cm- or mm-scale spatial resolution required to localize problem sources.

High resolution, remote imaging of temperature is routinely performed using infrared (IR) cameras for radiometry, exploiting the dependence of the blackbody radiation intensity at a given wavelength on temperature. This IR thermal imaging can be used e.g. to identify thermal leakage between indoor and outdoor environments [75]. Blackbody radiation is also emitted at microwave frequencies. Although the emission intensity from room temperature bodies is much lower at microwave frequencies than at IR frequencies, microwaves can penetrate through dielectric materials such as walls to assess spaces hidden to visible or IR frequencies, and can still provide spatial resolution on the mm- to cm-scale. Whilst promising, microwave thermometry for applications in the built-environment is at a much earlier stage of development than IR cameras and requires further development, with single-channel sensors of moderate sensitivity and spatial resolution reported [76, 77].

Water in all its forms (humidity, moisture, condensation, etc.) and its distributions are often key variables for the health of a building’s occupants [78–80]. Microwave radiometers are used in the large scale environment for remote measurement of atmospheric humidity. Water vapor can be identified from its distinct absorption peak at a frequency of 22.235 GHz in its microwave spectrum [81], and multi-frequency measurements can be used to detect liquid water (e.g. suspended microdrops). However, atmospheric measurements integrate over 100 s to 1000s of meters of air, and the research has not been done to determine if indoor microwave radiometry, integrating over much smaller volumes, would provide a sufficient signal for practical humidity measurements.

It may be more immediately practical to look for water in materials, such as walls with water leakage, which can both drive local humidity and host infectious agents themselves. Wet material can be indirectly detected with IR imaging, through the resulting change in surface temperature [75]. Direct remote sensing techniques for indoor water are less advanced, and would require research and development effort to become practical.

Microwave-based sensing is again promising, due to its through-wall imaging capability and ability to provide sufficient spatial resolution [82] (unlike radiation of longer wavelengths). Microwave sensors for water can be based on the scattering of microwaves generated by an external source, as discussed in this issue [83], with holographic techniques able to provide 3D reconstructions of scattering objects [84, 85]. In the short to medium term, single-channel point-sensors could be developed, with imaging performed by scanning the sensor. As a long-term aspiration, one could envisage a camera providing images of 3D moisture levels in real time, similar to temperature imaging with IR cameras. Schlieren and shadowgraph optics allow 3D imaging of air movements at distance [86]. It remains to be seen if these and/or other methods can be developed to noninvasively monitor the full range of airflows relevant to building occupants. A strength of microwaves are their ability to penetrate many building materials even though they are strongly absorbed by water and reflected by metals. Infrared has the advantage of better specific spectra for many materials of interest.

Real time carbon dioxide (CO_2_) imaging in the built environment seems a reasonable approach to become both practical and informative. The atmospheric concentration of CO_2_ is about 400 ppm (ppm), 0.04% and increasing at an annual rate of approximately 2-4 ppm per year [87]. Directly exhaled human breath is between 4 and 5% CO_2_, approximately 100 fold more than the atmosphere. Increased ambient CO_2_ correlates well with subjective reports of stuffy air [88] and local areas of increased concentration correlate well with increased airborne concentrations of *Mycobacterium tuberculosis* [89]. CO_2_ quantification is carried out with compact devices that measure concentration via specific IR spectral absorption. Wavelength-specific IR is used for earth [90] and extraterrestrial [91] quantification of atmospheric CO_2_. To our knowledge IR imaging of CO_2_ has not yet been used in the built environment. If there is clear motivation to do it, then it can be done. Real time IR imaging of CO_2_ might, for example, be used to direct a small fan into regions of high CO_2_ thereby dispelling patches of stagnant air in an energy efficient manner.

## Public health policymakers

The global health world is waking up to the challenges posed by respiratory infectious diseases outbreaks, with lessons being learned from high profile outbreaks covering coronaviruses such as SARS and MERS, and the ‘bird flu’ and ‘swine flu’ events from 2005 and 2009 [92]. The Ebola outbreak in west Africa across 2014 and 2015, although not primarily driven by respiratory transmission, has also triggered international and national level plans for dealing with communicable disease [93].

Powerful global health actors, that for example include the World Health Organization [94], Wellcome Trust [95], World Bank [96] and the Bill & Melinda Gates Foundation [97], have now developed action plans, protocols, policy documents and research programs that address some current needs and tentatively cover emerging and future priorities. However, we feel there is much they could still do in leading a conversation about the future of public health, and how interventions and technology can drive both subtle and radical transformations in this particular landscape.

Infections usually disproportionately affect the most vulnerable populations, such as children with severe malnutrition in resource-poor settings. An arguably lower-profile aspect of the 2030 Sustainable Development Goals, within the ‘no one left behind’ mantra, is the concept of healthy ageing [98]. With an increasingly ageing global population, we hypothesize that managing newly-geriatric populations in low- and middle-income settings will result in requirements for entire new medical and public health specialties in countries where currently few people live in to their 70s or older. An increasing number of older people, presenting with numerous morbidities (such as cardiovascular, musculoskeletal complications, and dementia), will therefore not be able to be cared for in a family home, and local equivalents of care homes will be set up. The elderly institutionalized remain vulnerable to infection. This will result in development of clinical practice, and ethical and legal, frameworks for patient management and research in vulnerable populations and these may best adopted from existing frameworks (such as the Mental Capacity Act in the UK), and adapted to suit local needs and cultures [99, 100].

Future public health preventive measures include more efficiently- targeted approaches to vaccination. The concept of transmissible vaccines is being widely considered, and initial theoretical research has suggested that even weakly transmissible vaccines could provide significant benefit in reductions of disease burden [101, 102]. The additional benefit of improved vaccination programs would be to slow the development of antimicrobial resistance [103, 104]. However, the potential negative consequences on vulnerable immunocompromised individuals, must be clearly considered. Public discussion concerning new vaccine programs has sometimes been politically charged [105].

## Conclusions

### A data-driven dilemma in the future of public health ethics

Public health, with reference to microbiology, utilizes several streams of data all of which are subject to improvements whose combined information have the potential to be synergistic: 1) identification and quantification of microbes, their locals of growth, metabolism, evolution, carriers, and 2) mapping the physical parameters that contribute to microbial survival and dissemination. 3) Patient and epidemiological reporting 4) Correlation indicators such as Google searches. New technologies will increase the temporal and spatial resolution of data collection and move closer to real time apprehension of the microbial environment. Higher resolution information will inspire architectural and social designs that aim to diminish detrimental human-pathogen encounters while enhancing those that may be beneficial. Personal and Precision Public Health have the potential to greatly reduce the spread of infections and the burden of antibiotic resistance both directly by spotting antibiotic resistance genes earlier and indirectly by decreasing the overall rate of primary infection. The displacement of antibiotic tolerant *C. difficile* with microbiomes introduced via fecal transplants are already a clinical application of introducing health-promoting microbiomes [106]. It remains to be seen if this is an isolated and specialized application or, more hopefully, a harbinger of a broader future.

The realization of Precision Public Health will require new interdisciplinary collaborations inclusive of sensor engineers, microbiologists (both medical and environmental), building engineers, architects and bioethicists. Focused multi-disciplinary training programs in high-income settings can lead the way for significant knowledge and skill transfer into low- and middle-income settings. Fine grained spatial and temporal mapping of physical and biological parameters along with reduced lag between data gathering and interpretation will progress toward real time analysis. Transformative benefits in personal care via personal and precision medicine are widely touted [107] but we assert that personal and Precision Public Health offer opportunities for impact at greater scale.

Ethical issues that encompass work, civil, and human rights need to be considered right from the very start of planning around Precision Public Health. The public must have a say in these future public health initiatives. Retrospective public and community engagement can lead significant and harmful loss of trust in public health, as demonstrated in the UK with the failed implementation of the care.data digital health program [108], and in Denmark where unwarranted anti-vaccine messages about the HPV vaccine overwhelmed a limited health promotion campaign [109].

In the event of infection with highly-infectious or serious pathogens, people- no one can say yet how many- may be excluded from their chosen work, travel, or even confined against their will based only on their predicted and unintended likelihood of spreading disease. It would be a new kind of sequence-based discrimination and the incriminating sequences would not even be their own. On the other hand, with appropriate intervention that may include confinement, the spread of contagion might be greatly diminished in many circumstances, with work and health of the entire group- especially the most immunologically vulnerable- greatly enhanced. Who could argue against increasing the safety of hospital workers and patients? Who could argue against greater microbiological safety and health for the crew of long term space flights who live at close quarters for months at a time [110]? If there were clear evidence for wider public health benefit, how could we not do this? Even in Typhoid Mary’s day (early 1900’s, See Table 1) asymptomatic carriers of the typhoid bacillus could be identified through individual stool samples. Present and foreseeable technologies make us all candidates for routine high resolution screening. Future approaches to sampling in Precision Public Health will ideally be unobtrusive but consequences, i.e. how the information is used, are part and parcel of generalized changes to the practical as well as philosophical aspects of privacy, individual rights, and free will [111] that merit as much forethought with all the relevant stakeholders as the technology.

**Table 1:** Typhoid Mary and Minority Report

The premise of “Minority Report” (a dystopian science fiction story by Phillip K. Dick (1956), made into a film (2002) and a television series (2015)) is “predictive policing” that allows authorities to forecast who will commit a crime. The “pre-offender” is preventatively detained or killed before any crime has actually taken place. Mary Mallon, better known as “Typhoid Mary” was an asymptomatic carrier of the typhoid fever bacillus whose unfortunate choice of occupation was to be a cook. Epidemiologists tracked her down, but she escaped surveillance, changed her name and again took up the only trade she knew. Repeatedly located by investigators who tracked down the shared source of multiple infections, she was eventually confined against her will with no prospect of release [113]. Finding and acting to prevent contagion from Typhoid Mary saved lives and reduced a significant local burden of enteric disease, even though it compromised her civil rights. The molecular version of “Minority Report” would be to prospectively identify Typhoid Mary rather than relying on reactive surveillance to follow the trail of disease backwards to find its source. Typhoid Mary is a singular and extreme case. It is also a century old. The moral calculus has not changed with more recent cases although the information-dissemination and decision-making machinery has, and not always for the better [114, 115]. By considering the role that information plays in more recent cases we try to consider how better and different information may inform future public health policies. Anticipated technical advances to prospectively screen carriers of transmissible disease universalize this possibility which is both an opportunity or a threat. This commentary article discusses enabling technologies and also considers implications for both public health and private rights.	

## Abbreviations

AMR: AntiMicrobial Resistance
BSE: Bovine Spongiform Encephalopathy
FACS: Fluorescence Activated Cell Sorting
Ghz: Gigahertz
HPV: Human Papilloma Virus
HVAC: Heating, Ventilation, and Air Conditioning
IoT: Internet of Things
IR: Infrared
MERS: Middle East Respiratory Syndrome
SARS: Severe Acute Respiratory Syndrome
VOC: Volitile Organic Compounds
